# Retinal ganglion cell degeneration correlates with hippocampal spine loss in experimental Alzheimer’s disease

**DOI:** 10.1186/s40478-020-01094-2

**Published:** 2020-12-07

**Authors:** Ryan J. Bevan, Tim R. Hughes, Pete A. Williams, Mark A. Good, B. Paul Morgan, James E. Morgan

**Affiliations:** 1 School of Optometry and Vision Sciences, Cardiff University, Cardiff, CF24 4HQ UK; 2 UK Dementia Research Institute, Cardiff University, Cardiff, UK; 3 Systems Immunity Research Institute, Cardiff University, Cardiff, UK; 4 Department of Clinical Neuroscience, Division of Eye and Vision, St. Erik Eye Hospital, Karolinska Institutet, Stockholm, Sweden; 5 School of Psychology, Cardiff University, Cardiff, UK

**Keywords:** Alzheimer’s disease, Retinal ganglion cells, Dendritic spines, DiOlistic labelling, Sholl analysis, Synaptic pruning

## Abstract

Neuronal dendritic and synaptic pruning are early features of neurodegenerative diseases, including Alzheimer’s disease. In addition to brain pathology, amyloid plaque deposition, microglial activation, and cell loss occur in the retinas of human patients and animal models of Alzheimer’s disease. Retinal ganglion cells, the output neurons of the retina, are vulnerable to damage in neurodegenerative diseases and are a potential opportunity for non-invasive clinical diagnosis and monitoring of Alzheimer’s progression. However, the extent of retinal involvement in Alzheimer’s models and how well this reflects brain pathology is unclear. Here we have quantified changes in retinal ganglion cells dendritic structure and hippocampal dendritic spines in three well-studied Alzheimer’s mouse models, Tg2576, 3xTg-AD and APP^NL-G-F^. Dendritic complexity of DiOlistically labelled retinal ganglion cells from retinal explants was reduced in all three models in an age-, gender-, and receptive field-dependent manner. DiOlistically labelled hippocampal slices showed spine loss in CA1 apical dendrites in all three Alzheimer’s models, mirroring the early stages of neurodegeneration as seen in the retina. Morphological classification showed that loss of thin spines predominated in all. The demonstration that retinal ganglion cells dendritic field reduction occurs in parallel with hippocampal dendritic spine loss in all three Alzheimer’s models provide compelling support for the use of retinal neurodegeneration. As retinal dendritic changes are within the optical range of current clinical imaging systems (for example optical coherence tomography), our study makes a case for imaging the retina as a non-invasive way to diagnose disease and monitor progression in Alzheimer’s disease.

## Introduction

Alzheimer’s disease (AD) is the underlying cause of up to 70% of all dementias and estimated to afflict 40 million people worldwide by 2050 [32, 36]. AD pathology is characterised by extracellular amyloid plaques, neurofibrillary tangles, neuroinflammation and synaptic loss that ultimately result in severe brain atrophy [14, 37, 42, 49]. Synaptic dysfunction is the strongest correlator to AD severity and a target for therapeutic intervention [49, 51]. Currently, the detection of early neurodegeneration is challenging and relies on insights from AD animal models that recapitulate aspects of the disease.

Visual impairment has been reported as an early symptom in some individuals diagnosed with neurodegenerative disease, including AD [11, 25]. Visual deficits can include reduced acuity, visual field defects, changes in contrast sensitivity, impaired object recognition, and delayed visual processing [30]. While of interest, these observations have not pinpointed specific deficits in the visual pathway that consistently account for a reduction in visual performance. However, retinal changes have received little attention. The retina has been proposed as a surrogate for in vivo detection and monitoring AD-related changes in the central nervous system (CNS) since the retina can be considered an extension of the brain and is accessible for imaging [28, 29, 38]. AD retinal pathology resembles that found in the brain, including neuronal cell loss, particularly of retinal ganglion cells (RGCs) [8]. However, loss of RGCs, the output neuron of the retina, and resulting thinning of the retinal nerve fibre layer (RGC axons) suggests that these could be a meaningful index of cognitive decline and a feature in AD [31, 45].

RGCs may be particularly susceptible to damage in the context of AD because of their intraocular unmyelinated axons, high energy demand and complex dendritic arbours [20]. The morphology of the RGC dendritic field and the disruption of glutamatergic synapses along the dendrites associate with neuronal function and dendritic degeneration correlates with visual decline [33]. RGC dendritic pruning is characterised by the loss of dendritic complexity and is a feature in retinal neurodegeneration models [52, 55]. The underlying mechanism of dendritic loss is unclear; however, it is an important characteristic in the early stages of neurodegeneration. We have previously reported that RGC dendritic degeneration is an early pathological event in the Tg2576 AD mouse model compared to littermate wildtype controls [54]. However, it is not known whether these findings are reproducible across other AD mouse models, are present at different time points in disease pathogenesis, and, whether RGC dendritic loss correlates with degenerative events in the CNS in the models.

To address these issues, we characterised the RGC dendritic morphology in amyloid overexpressing (Tg2576, 3xTg-AD) and knock-in (APP^NL-G-F^) AD mouse models. Using DiOlistic labelling, a technique that is not contingent on cell health, RGCs were morphologically assessed and compared to the dendritic spine densities in the hippocampus in the same mice [18, 41, 52]. Our findings demonstrate consistent and progressive RGC degeneration from early in the disease process in both overexpressing and knock-in AD models. Retinal changes also correlated with a reduction in hippocampal spine densities in each of the models. The findings support the assessment of retinal changes to detect early-stage neurodegeneration and monitor disease progression in models and man.

## Methods

### Animals

All animal experiments complied with the UK Animals Scientific Procedures Act 1986 and ethical guidelines at Cardiff University. Experiments were carried out under the authority of UK Home Office licences 30/3220, P8159A562 and 30/03313. Mice were group-housed where possible in environmentally enriched cages with food and water ad libitum in a 12-h light/dark cycle. Tg2576 (In-house line, (strain: B6; SJL-Tg(APPSWE)2576Kha), 3xTg-AD (The Jackson Laboratory, (strain 34830-JAX: B6; 129-Tg(APPSwe, tauP301L)1Lfa Psen1^tm1Mpm/Mmjax^)) and APP^NL-G-F^ (RIKEN, Japan) (strain: App^tm3.1Tcs^/App^tm3.1Tcs^)) mouse models were analysed with Tg2576 compared to wildtype littermate controls (WT Control) and the 3xTg-AD and APP^NL-G-F^ models compared to C57BL/6J (B6/J Control, in house colony). It was not possible to compare 3xTg-AD and APP^NL-G-F^ mice to littermate controls; instead, we compared our observations to C57BL6/J mice (B6/J Controls) since the APP^NL-G-F^ model are based on the same background. Littermate controls for the 3xTg-AD model are not available due to the mixed background and shotgun approach used to generate this model. The Jackson Laboratory offer an approximate control (B6129SF2/J, Stock No: 101045) for the 3xTg-AD strain, however, this strain is known to develop other confounding features. Both sexes were analysed for B6/J Control, 3xTg-AD and APP^NL-G-F^; however, only males were available for the Tg2576 model. Table 1 provides details on the number of mice, RGCs and hippocampal dendritic segments analysed from each model at 6–7 months and 12 months. The specific number of mice, RGCs and hippocampal dendritic segments are included where appropriate with the associated figures.

**Table 1:** Number of mice, RGCs and hippocampal dendritic segments across all models analysed

Model	Age analysed: 6–7 months			Age analysed: 12 months		
	Mice (n)	RGCs (n)	Hippocampal dendritic segments (n)	Mice (n)	RGCs (n)	Hippocampal dendritic segments (n)
WT Control	X	X	X	7	74	71
Tg2576	X	X	X	8	58	85
B6/J Control	6	58	47	10	114	106
3xTg-AD	9	99	77	12	144	223
APP^NL-G-F^	7	94	46	6	80	130
Total	22	251	170	43	470	615

*WT* wildtype, *RGCs* retinal ganglion cells

### Tissue harvest

For retinal analysis, eyes were enucleated and transferred to chilled 4 °C HBSS (Hank’s Balanced Salt Solution), punctured with a needle at the limbus and the cornea, lens, vitreous and sclera removed. Retinal explants were flat-mounted ganglion cell layer up. For hippocampal analysis, whole brains were removed and cut sagittally in chilled 4 °C HBSS. In one hemisphere, parasagittal hippocampal regions were sliced (200 µm) in a septal to temporal progression using a McIlwain tissue chopper. The total time for retinal explant preparation from both eyes and hippocampal slicing in a single mouse was less than 10 min, since time is critical for DiOlistic labelling efficiency.

### DiOlistic preparation and neuronal labelling

Fresh retinal explants and hippocampal slices prepared as above were transferred to glass slides and subjected to gene gun (BioRad) DiOlistic labelling of neurons [10, 47, 50]. In brief, Ethylene tetrafluoroethylene (ETFE) tubing was coated with 1.67 µm tungsten particles coated with 1,1′-Dioctadecyl-3,3,3′,3′-Tetramethylindocarbocyanine Perchlorate (DiI, Life Technologies) and 3,3′Dioctadecloxacarbocyanine Perchlorate (DiO, Life Technologies). Dye coated ETFE ‘bullets’ were cut at 1.2 cm, loaded into the gene gun and fired onto the slides at a pressure of 100–120 psi through a 3.0 µm membrane (BD Falcon 3.0 µm, BD Biosciences). Slides were then immersed in Neurobasal-A medium (Life Technologies) for 20 min at 37 °C with 5% CO_2_. Dye staining was checked under a fluorescence microscope to confirm neuron labelling then slides were fixed in 4% PFA (paraformaldehyde) for a minimum of 30 min, with subsequent nuclei labelling with Hoechst 33258. Slides were coverslipped with FluorSave and stored in the dark at 4 °C until analysis.

### Confocal imaging

All confocal images were captured using a Leica SP8 Lightning confocal microscope. RGCs were identified by their morphology and relative position in the retina, i.e. in the ganglion cell layer, characteristic dendritic field and a clear axon projecting towards the optic nerve. RGCs with overlapping dendritic fields that prevented single-cell delineation were excluded from the analysis. Dendritic arbours were imaged using 20 × objective (z-axis, interval 1 µm). Hippocampal dendrites (CA1 field) were selected based on the minimal overlap of adjacent cells. CA1 apical dendritic spines were imaged at 63 × objective (z-axis, interval 0.2 µm) using Lightning Deconvolution (Leica). Typically, each hippocampal slice yielded at least one CA1 neuron suitable for imaging; 10–15 images/neurons were imaged per mouse.

### Neuronal reconstruction

All confocal images were analysed using Imaris software (version 9.2, Bitplane, Zurich, Switzerland). RGC dendritic fields and hippocampal dendrites were reconstructed using the Filament Tracer module with default thresholding based around a ‘region of interest,’ i.e. the dendritic field. All dendritic arbours were automatically traced within the software with any incorrect tracing, e.g. adjacent cells or dye debris manually removed. The associated statistical parameters were exported from Imaris for data analysis with Sholl analysis used for a measure of dendritic complexity (at set intervals of 10 µm). Dendritic spines were reconstructed using the Filament tracer in the same process as retinal ganglion cells; however smaller ‘regions of interest’, i.e. dendritic segments typically of 30 µm were analysed. Spines were detected using the Filament Tracer module and classified using the SpineClassifer MATLAB extension. Spine types were distinguished on the basis of spine length and spine head size.

### Retinal ganglion cell receptive field classification

Retinal ganglion cells were classified based on their dendritic lamination into the inner plexiform layer [1, 53]. Dendrites projecting into the sublamina b (closest to the ganglion cell layer) were termed ON-centre; conversely, dendrites projecting into the sublamina a were termed OFF-centre [56].

### Nuclei counts

The number of nuclei in the ganglion cell layer were quantified from Hoechst labelling from the DiOlistic RGC confocal images. All RGC images with Hoechst 33258 labelling were quantified using the Spots function in Imaris and averaged to obtain a nuclei ganglion cell layer count per animal. Parameters were kept consistent for all quantifications.

### Statistics

Data are represented as individually reconstructed retinal ganglion cells and dendritic segments for spine densities collected for each group. Statistical analyses were performed using GraphPad Prism software (version 8) with the statistical tests and details of data reported in each figure legend where appropriate. Data were analysed using the Mann–Whitney U test, 1-way analysis of variance (Kruskal–Wallis test) using Dunn multiple comparisons post-test or two-way ANOVA, as appropriate. Associations between pathological variables were tested by Spearman analysis. Significant results are denoted as follows: * = *p *< 0.05, ** = *p *< 0.01, *** = *p *< 0.001, **** = *p* < 0.0001.

## Results

### Retinal ganglion cell degeneration is a common feature of the Tg2576, 3xTg-AD and APP^NL-G-F^ AD mouse models

**Fig. 1:**
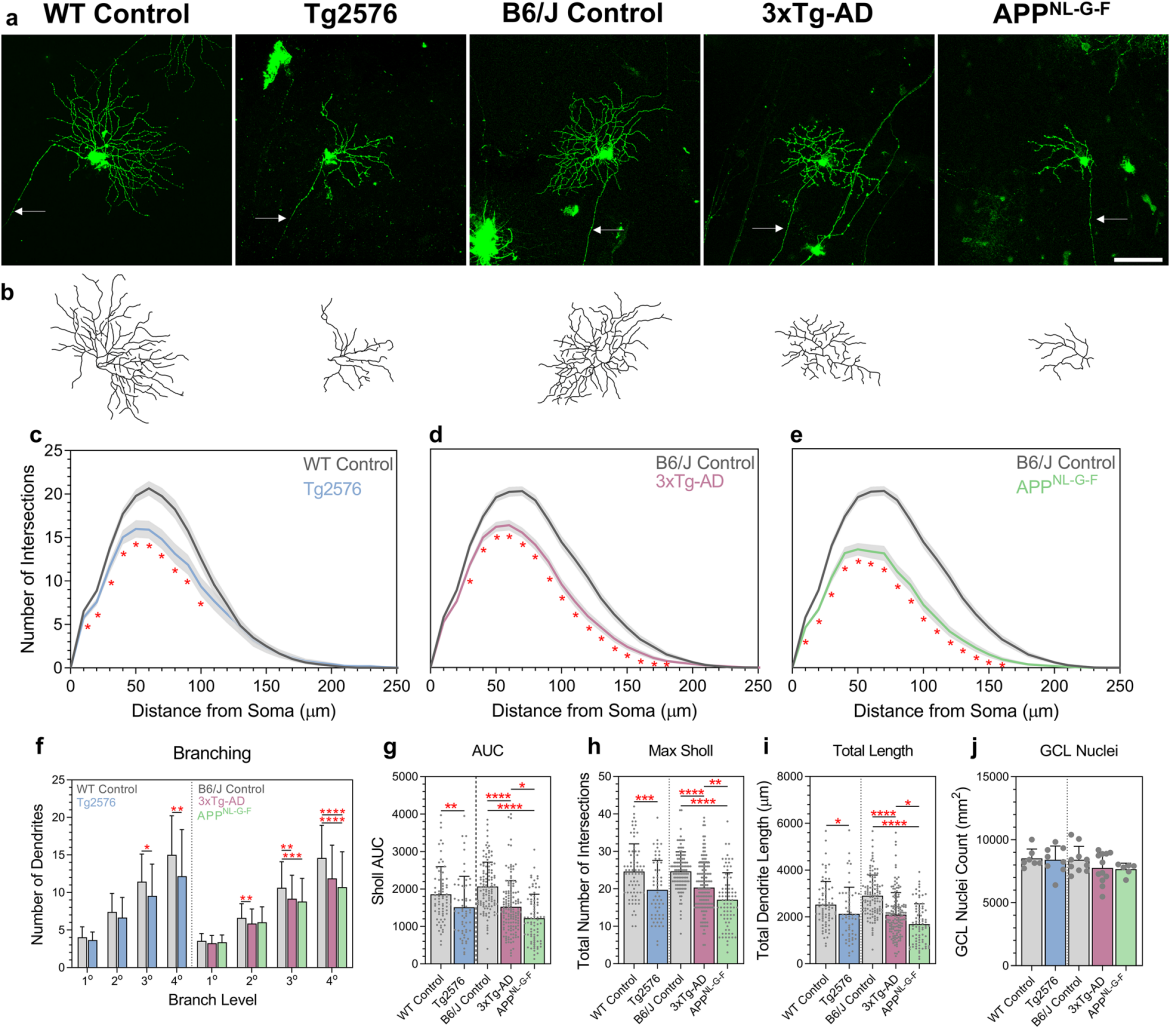
RGC dendritic loss at 12 months in Tg2576, 3xTg-AD and APP^NL-G-F^ mice. **a** Representative confocal images of RGCs from WT Control, Tg2576, B6/J Control, 3xTg-AD and APP^NL-G-F^. Arrows indicate axon. Scale bar 100 µm. **b** Corresponding dendritic field reconstructed in Imaris. **c**–**f** Sholl analysis of all reconstructed RGCs comparing single models to show significance. Line represents mean of group at each interval, shaded error zones represent ± SEM. Two-tailed Mann–Whitney U test at each interval distance, *p < 0.05, **g** number of dendrites at each branching level proximal to the cell body. Error bars correspond to SD. **h** Area under the Sholl curve. **i** Maximum number of Sholl intersections. **j** Total length of all dendrites. **k** Number of Hoechst-positive nuclei in the GCL. Column Scatterplots **g** bars represent mean from individual cells and error bars correspond to SD. Column Scatterplots **h**–**j** data points represent individual cells and error bars correspond to SD. Column Scatter plot **k** data points represent individual mice and error bars correspond to SD. WT Control: *n* = 74 cells (7 male mice), Tg2576: *n* = 58 cells (8 male mice), B6J Control: *n* = 114 cells (10 mice, 5 male/5 female), 3xTg-AD: *n* = 144 cells (12 mice, 6 male/6 female) and APP^NL-G-F^: *n* = 80 cells (6 mice, 3 male/3 female). *p < 0.05, **p < 0.01, ***p < 0.001, ****p < 0.0001. All mice aged 12 months. *WT* wildtype, *AUC* area under the Sholl curve, *GCL* ganglion cell layer

**Fig. 2:**
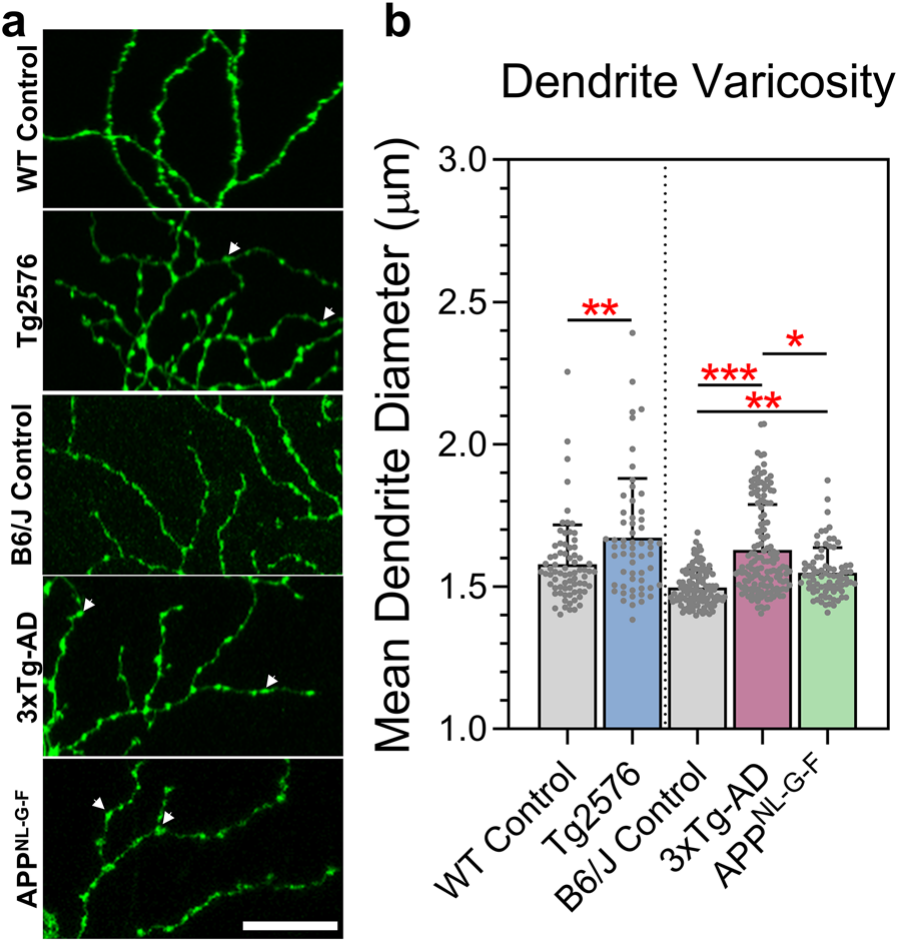
RGC dendritic diameter increases in AD mice. **a** Representative confocal images of RGC dendrites from WT Control, Tg2576, B6J Control 3xTg-AD and APP^NL-G-F^. Arrows indicate beading and swelling of dendrites. Scale bar 25 µm. **b** Dendrite varicosity, assessed by measurement of dendrite diameter. Column Scatterplots: data points represent individual cells and error bars correspond to SD. WT Control: *n* = 74 cells (7 male mice), Tg2576: *n* = 58 cells (8 male mice), B6J Control: *n* = 114 cells (10 mice, 5 male/5 female), 3xTg-AD: n = 144 cells (12 mice, 6 male/6 female) and APP^NL-G-F^: *n* = 80 cells (6 mice, 3 male/3 female). All mice aged 12 months. *p < 0.05, **p < 0.01, ***p < 0.001, ****p < 0.0001

We first sought to determine whether RGC dendritic degeneration occurred in Tg2576, 3xTg-AD and APP^NL-G-F^ AD mouse models at 12 months, a time point when these models have been demonstrated to have hippocampal and behavioural degenerative changes that are indicative of AD-like pathology. Sholl analysis demonstrated significant reductions in dendritic complexity in all three AD models compared to respective age-matched wildtype control mice (littermate controls for Tg2576 mice and B6/J controls for 3xTg-AD and APP^NL-G-F^ mice) (Fig. 1a–e). Sub-analysis, according to dendrite branch hierarchy, showed preservation of primary dendrites across the three AD models. However, 3xTg-AD RGCs showed significant secondary dendritic loss, and all models showed tertiary and quaternary dendritic pruning (Fig. 1f). Overall the area under the Sholl curve (AUC; a global measure of dendritic complexity) showed significant pruning in all three AD models, with the greatest dendritic loss observed in the APP^NL-G-F^ retina (Fig. 1g). These reductions were matched by a decrease in the peak of the Sholl curve and the total dendrite length (Fig. 1h–i). Notably, the dendritic changes occurred in the absence of significant cell loss in the ganglion cell layer (GCL, Fig. 1j). We also observed dendritic varicosities (swelling of the dendrites) which coincided with an increased diameter of the dendrites situated around and after the peak of the Sholl curve (branching levels 4–7) in RGCs from all AD model mice (Fig. 2).

**Fig. 3:**
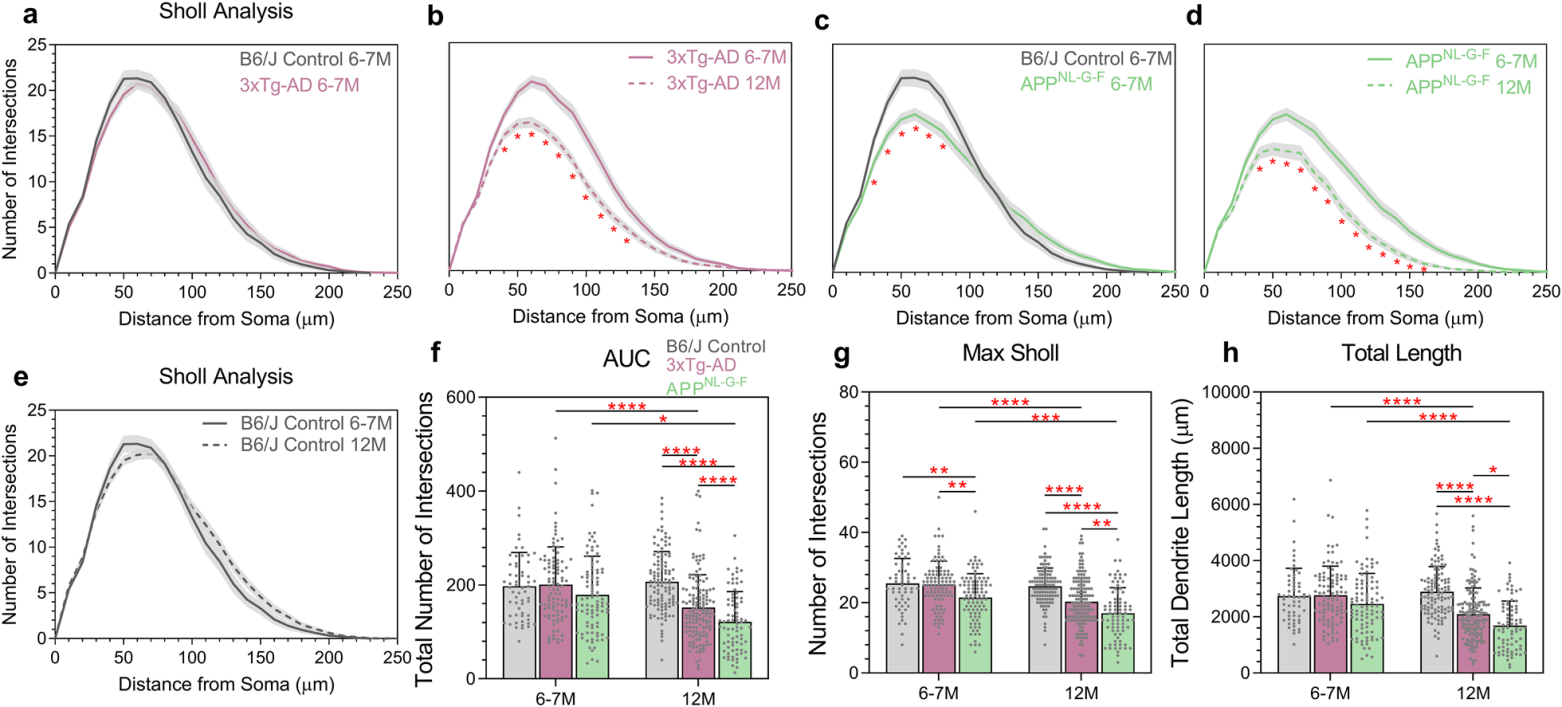
RGC dendritic loss progresses with age in 3xTg-AD and APP^NL-G-F^ mice. **a**–**e** Sholl analysis of all reconstructed RGCs from B6J Control, 3xTg-AD and APP^NL-G-F^ at 6–7 and 12 months comparing single models to show significance. Line represents mean of group at each interval, shaded error zones represent ± SEM. Two-tailed Mann–Whitney U test at each interval distance, *p < 0.05. **f** Area under the Sholl curve. **g** Maximum number of Sholl intersections. **h** Total length of all dendrites. Column Scatterplots **f**–**h** data points represent individual cells and error bars correspond to SD. B6J Control 6–7 M: *n* = 58 cells (6 mice, 6 male), B6/J Control 12 M: *n* = 114 cells (10 mice, 5 male/5 female), 3xTg-AD 6–7 M: *n* = 99 cells (9 mice, 9 male), 3xTg-AD 12 M: *n* = 144 cells (12 mice, 6 male/6 female), APP^NL-G-F^ 6–7 M: *n* = 94 cells (7 mice, 4 male/3 female mice) and APP^NL-G-F^ 12 M: *n* = 80 cells (6 mice, 3 male/3 female). *p < 0.05, **p < 0.01, ***p < 0.001, ****p < 0.0001. *WT* wildtype, *AUC* area under the Sholl curve

We were able to assess RGC dendritic structure in 6–7-month and 12 month 3xTg-AD, APP^NL-G-F^ and B6/J Control mice (Fig. 3). In 3xTg-AD mice at 6–7 months, RGCs showed no excess dendritic pruning compared to B6/J Control; however, at 12 months 3xTg-AD mice demonstrated significantly greater dendritic loss compared to controls (Fig. 3a, b). In comparison to B6/J Control, APP^NL-G-F^ mice at 6–7 months showed significantly greater RGC dendritic degeneration with a reduced Sholl profile corresponding to the highest branched areas of the dendritic field; comparison with APP^NL-G-F^ mice at 12 months showed significantly increased dendritic loss with age (Fig. 3c, d). In B6/J Control mice, RGC dendritic complexity was not different between 6-7 months and 12 months (Fig. 3e). These findings were also supported by quantitative assessments of the Sholl AUC, the peak of the Sholl intersections, and the total dendritic length (Fig. 3f–h) which demonstrate that increased RGC dendritic atrophy occurs in AD model mice and progressively worsens with age.

### ON-centre specific RGCs are more affected in the AD model mouse retina

**Fig. 4:**
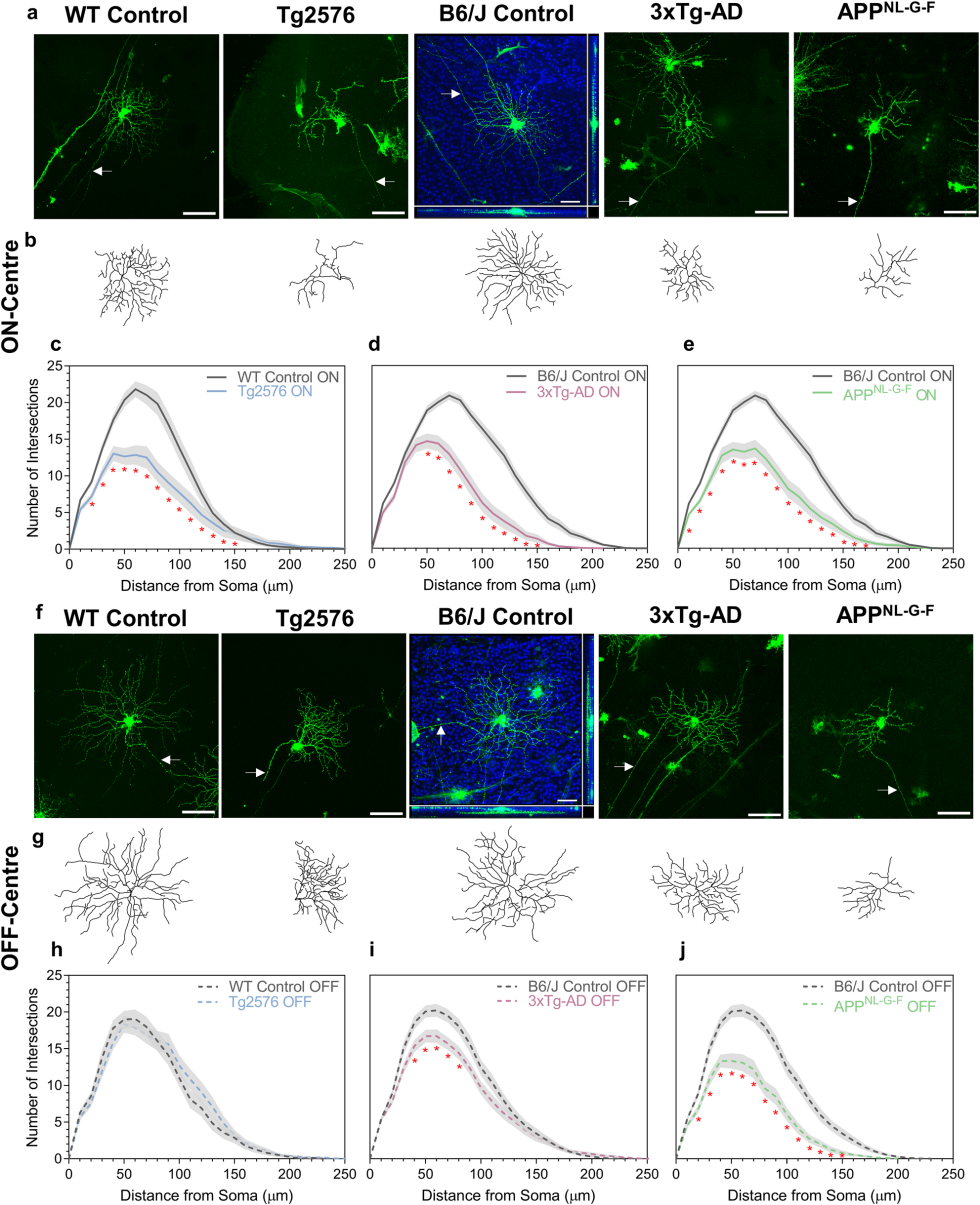
RGC receptive field classification impacts on dendritic loss. **a**–**b** Representative confocal images of ON-centre RGCs from WT Control, Tg2576, B6J Control 3xTg-AD and APP^NL-G-F^ with corresponding Imaris reconstructions. Arrows indicate axons. Scale bar 100 µm. **c**–**e** Sholl analysis of reconstructed ON-centre RGCs comparing single models to show significance. Two-tailed Mann–Whitney U test at each interval distance, *p < 0.05. **f**–**g** Representative confocal images of OFF-centre RGCs from WT Control, Tg2576, B6J Control, 3xTg-AD and APP^NL-G-F^ with corresponding Imaris reconstructions. Arrows indicate axons. Scale bar 100 µm. **h**–**j** Sholl analysis of reconstructed OFF-centre RGCs comparing single models to show significance. Two-tailed Mann–Whitney U test at each interval distance, *p < 0.05. Sholl analysis line represents mean of group at each interval, shaded error zones represent ± SEM. WT Control ON: *n *= 43 cells, WT Control OFF: *n* = 31 cells (7 male mice) Tg2576 ON: *n* = 30 cells, Tg2576 OFF: *n* = 28 cells (8 male mice); B6/J Control ON: *n* = 56 cells, B6/J Control OFF: *n* = 58 cells (10 mice, 5 male/5 female); 3xTg-AD ON: *n* = 69 cells, 3xTg-AD OFF: *n* = 75 cells (12 mice, 6 male/6 female); APP^NL-G-F^ ON: *n* = 38 cells, APP^NL-G-F^ OFF: *n* = 41 cells (6 mice, 3 male/3 female). All mice aged 12 months. *WT* wildtype

We next investigated whether RGCs changes were influenced by RGC type. RGCs with dendritic lamination in inner plexiform layer (IPL) sublamina *b* are classified as ON-centre and those with dendrites in sublamina *a* are classified as OFF-centre. RGCs were compared across all three AD models at 12 months (Fig. 4). Significant ON-centre RGC dendritic loss was present in all three AD models (Fig. 4a–e). APP^NL-G-F^ and, to a lesser degree 3xTg-AD mice demonstrated significant dendritic loss compared to controls in OFF-centre RGCs; a non-significant trend of increased dendritic loss in the OFF-centre RGCs was seen in Tg2576 mice compared to controls (Fig. 4f–j). Collectively, these data show a greater susceptibility for ON-centre RGC degeneration across the three AD models.

### Retinal ganglion cell dendritic impairment is more prominent in male AD mice

**Fig. 5:**
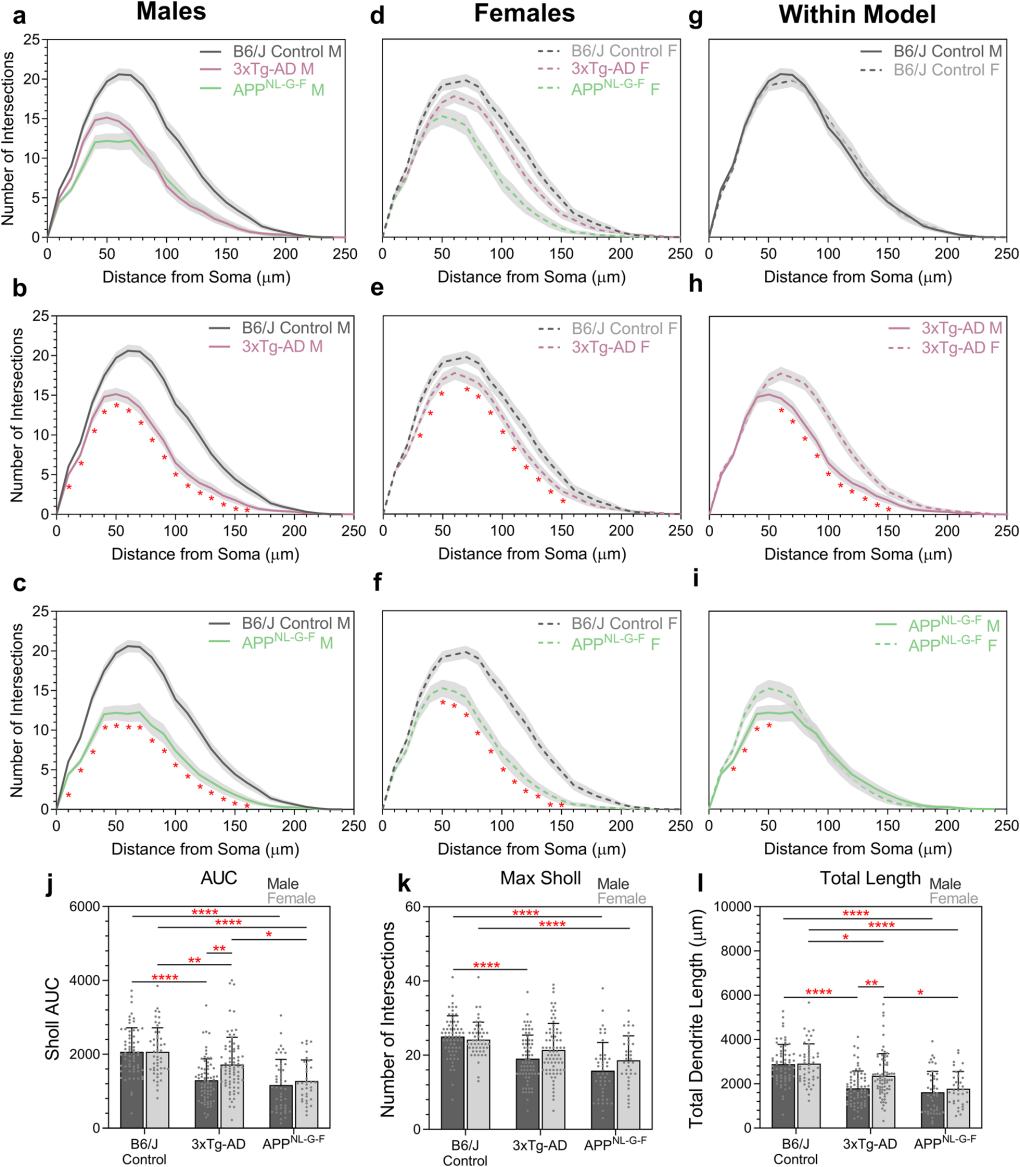
Gender influences the extent of RGC dendritic impairment at 12 months in the 3xTg-AD and APP^NL-G-F^ mice. **a**–**i** Sholl analysis of reconstructed RGCs from male and female mice compared to B6/J Control and within each model. **a** represents all the male data, **d** represents all the female data, **b**–**c**, and **e**–**i** compare single models to show significance. Two-tailed Mann–Whitney U test at each interval distance, *p < 0.05. Sholl analysis line represents mean of group at each interval, shaded error zones represent ± SEM. **j** Area under the Sholl curve. Two-way ANOVA: Interaction: F (2, 332) = 3.629, *p *= 0.0276; Genotype: F (2, 332) = 42.38, *p *< 0.0001; Gender: F (1, 332) = 5.701, *p *= 0.0175. **k** Maximum number of Sholl intersections. Two-way ANOVA: Interaction: F (2, 332) = 2.490, *p *= 0.0845; Genotype: F (2, 332) = 32.72, *p *< 0.0001; Gender: F (1, 332) = 4.182, *p *= 0.0416. **l** Total length of all dendrites. Two-way ANOVA: Interaction: F (2, 332) = 3.213, *p *= 0.0415; Genotype: F (2, 332) = 47.40, *p *< 0.0001; Gender: F (1, 332) = 6.137, *p *= 0.0137. Column Scatterplot **j**–**l** data points represent individual cells and error bars correspond to SD. B6J Control male: *n* = 64 cells (5 mice), B6J Control female: *n* = 50 cells (5 mice); 3xTg-AD male: *n* = 69 cells (6 mice), 3xTg-AD female: *n* = 75 cells (6 mice); APP^NL-G-F^ male: n = 45 cells (3 mice), APP^NL-G-F^ female: n = 35 cells (3 mice). All mice aged 12 months. *p < 0.05, **p < 0.01, ***p < 0.001, ****p < 0.0001. *AUC* area under the Sholl curve

Since gender-related differences in susceptibility have been reported in AD models, we next determined whether this was a factor influencing the degree of RGC dendritic pruning in the 3xTg-AD and APP^NL^^-G-F^ models where sufficient mice of both genders were available for analysis. Both male and female 3xTg-AD and APP^NL-G-F^ models demonstrated significant RGC dendritic loss compared to gender-matched B6/J Control (Fig. 5a–i). There were no morphological differences in RGCs between male and female B6/J Control mice (Fig. 5g). In contrast, we observed increased degeneration in RGCs from male compared to female mice in both the 3xTg-AD and APP^NL-G-F^ models, the former exhibiting the largest difference between genders (Fig. 5h, i). These findings were supported by quantitative measures of the Sholl AUC (Fig. 5j), the peak of the Sholl curve (Fig. 5k), and total dendrite length (Fig. 5l).

### Retinal pathology reflects hippocampal dendritic spine loss in the AD models

**Fig. 6:**
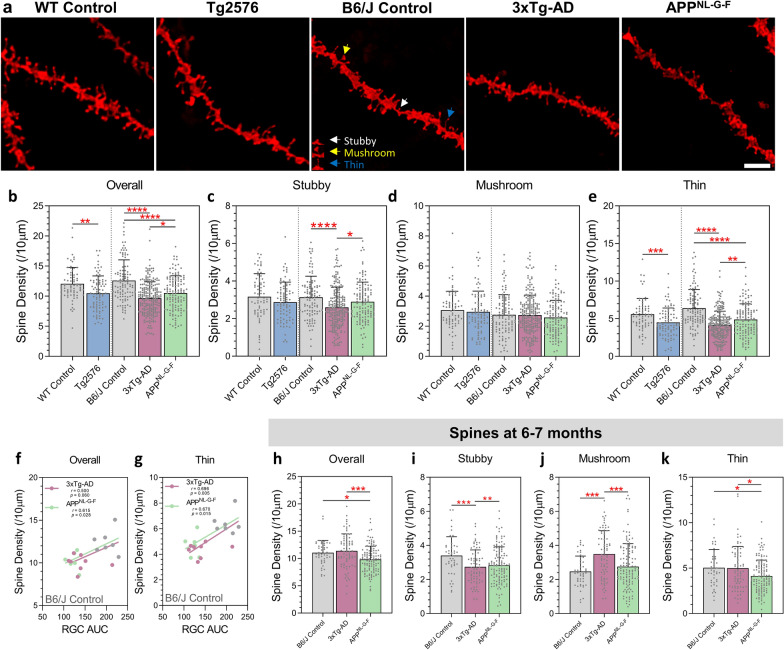
Hippocampal dendritic spine loss correlates with RGC dendritic changes in Tg2576, 3xTg-AD and APP^NL-G-F^ mice. **a** Representative confocal images of DiI labelled dendritic segments of CA1 segments from WT Control, Tg2576, B6J Control, 3xTg-AD and APP^NL-G-F^. Scale bar 5 µm. Spine densities were analysed from dendritic segments of at least 30 µm **b** Overall spine density. **c** Stubby spine density. **d** Mushroom spine density. **e** Thin spine density. **f** Total number of dendritic spines compared to RGC AUC per mouse for 3xTg-AD and APP^NL-G-F^ models (Spearman correlation). **g** Total number of thin dendritic spines compare to RGC AUC per mouse for 3xTg-AD and APP^NL-G-F^ models (Spearman correlation). Data represented as average per mouse. **g** Column Scatterplot data points represent individual cells and error bars correspond to SD. Overall (**h**), stubby (**i**), mushroom (**j**) and thin (**k**) spine density from B6/J Control, 3xTg-AD and APP^NL-G-F^ mice at 6-7 months. For 12 month data: WT Control *n* = 71 dendrites (5 male mice), Tg2576: *n* = 85 dendrites (8 male mice), B6/J Control: *n* = 106 dendrites (7 mice, 4 males/3 females), 3xTg: *n* = 223 dendrites (14 mice, 6 males/8 females), APP^NL-G-F^: *n* = 130 dendrites (6 mice, 3 males/6 females). For 6–7 month data: B6/J Control: *n* = 47 dendrites (5 female mice), 3xTg: *n* = 77 dendrites (5 male mice), APP^NL-G-F^: *n* = 46 dendrites (7 mice, 4 males/3 females). All mice aged 12 months. *p < 0.05, **p < 0.01, ***p < 0.001, ****p < 0.0001. *WT* wildtype, *AUC* area under Sholl curve

To determine the relationship between retinal and CNS (hippocampal) neurodegeneration, we measured, in the same mice, dendritic spines in the hippocampus of Tg2576, 3xTg-AD and APP^NL-G-F^ mice at 12 months (Fig. 6a). All three AD models displayed overall reductions in spine density on CA1 hippocampal neurons compared to respective wildtype controls, with 3xTg-AD mice the most severely affected (Fig. 6b). Dendritic spines can be subdivided based on morphological differences that reflect the functional diversity of the synapses [39]. Classifying the dendritic spines based on morphology revealed differences between the models. Thin spines showed a significant reduction in all models, while only 3xTg-AD mice demonstrated a significant reduction in the number of stubby spines, and mushroom spines were unaffected across all models (Fig. 6c–e). Loss of spines, significantly correlated with RGC dendritic loss in the 3xTg-AD and APP^NL-G-F^ mice when separated on morphology, thin spines showed the best correlation (Fig. 6f–g). We also analysed CA1 spine densities in B6/J Control, 3xTg-AD and APP^NL-G-F^ mice between the ages of 6–7 months (Fig. 6h–k). At this age, overall spine density was only reduced in APP^NL-G-F^ mice; again thin spines were the most affected. In the 3xTg-AD mice, despite no apparent overall spine reduction, the number of stubby spines was significantly decreased whilst the density of mushroom spines was increased compared to B6/J Controls. These data demonstrate that hippocampal spine loss is a consistent feature in all models and correlates with RGC changes.

## Discussion

Dendritic and spine loss are among the earliest events in neurodegeneration. While regions such as the hippocampus and cortex, are primary sites of pathology in AD, other parts of the CNS, including the retina, are affected [15, 26]. AD-relevant changes in the retina in AD models and human disease offer the prospect of non-invasive monitoring through in vivo imaging of retinal pathology as a tool to diagnose disease, monitor progression and test the efficacy of AD treatments; however, to date, reports of retinal pathology in humans and animal models have been few and inconsistent [5]. Justino et al. [22] suggested no retinal dysfunction based on normal scotopic and photopic flash electroretinograms (fERGs) and oscillatory potentials (OPs) in AD cases. While others report the absence of amyloid deposits and tau pathology in the retinas of post mortem AD cases, despite the detection in the brain [16]. In the retina of APP/PS1 mice, Chidlow et al. [5] found no relevant retinal pathology despite the presence of robust brain amyloid pathology, however in the same model Koronyo-Hamaoui et al. [23] report the in vivo detection of amyloid plaques in the retina that accumulated with disease progression. We have previously reported a preliminary study that documented reduced RGC dendritic complexity in the Tg2576 AD mouse model [54]; RGC dendritic integrity was reduced at 14 months. Here we have confirmed and extended these findings; we found significant RGC dendritic loss in Tg2576 mice compared with age-matched littermate controls at 12 months that occurred alongside observed hippocampal spine loss in the same animals.

In order to explore whether this observation could be general in nature, we next examined retinal and hippocampal pathology in two other mouse AD models, the widely used 3xTg-AD model (transgenic expression of three risk genes; APP Swedish, MAPT P301L, and PSEN1 M146V) and the recently described APP^NL-G-F^ model (knock-in of a humanized Aβ region including the Swedish “NL”, the Iberian “F”, and the Arctic “G” mutations). It was not possible to compare 3xTg-AD mice to littermate controls because of the complex background of this strain as has been noted by The Jackson Laboratory (https://www.jax.org/strain/101045), therefore we pragmatically chose to use C57BL6/J mice (B6/J) as controls. Animal breeding constraints prevented the use of littermate controls for the APP^NL-G-F^ mice; these are on the B6/J background so B6/J mice were used as controls. We selected DiOlistic labelling as a technique for measurement of dendritic complexity because the DiOlistic labelling of individual cells is not contingent on the health of the cell [19]. Using other methods such as the expression of fluorescent proteins can be problematic in chronic disease models since healthier neurons may show higher levels of expression, reducing sensitivity for detecting degenerating neurons. We have previously reported this effect when comparing degeneration in retinal ganglion cells labelled with YFP compared with DiI in a chronic murine glaucoma model [52]. Using multiple morphological parameters, we observed significant RGC dendritic loss compared to B6/J controls at 12 months in the 3xTg-AD and APP^NL-G-F^ models, with the greatest severity seen in the APP^NL-G-F^ mice. At 6–7 months there was no significant loss of RGC dendrites in the 3xTg-AD mice; in contrast, APP^NL-G-F^ mice showed significant RGC dendritic loss even at this early age, though not to the extent seen at 12 months in this model, confirming disease progression with age in both models. The identification of RGC dendritic loss may be reflected in a reduction in the thickness of the retinal layers. Although we did not measure this, others have reported that 3xTg-AD mice display thinning of the retinal sublayers, including the GCL and IPL, which correlate with changes occurring the brain [7]. We also observed significant swelling and beading of the dendrites in the RGCs from AD models, possibly indicating mitochondrial dysfunction [12]. Of note, there was no significant reduction in number of nuclei in the ganglion cell layer implying that the number of RGC’s were unaffected. In all three models, RGC dendritic loss occurred in parallel with hippocampal dendritic spine loss; these correlated strongly in the 3xTg-AD and APP^NL-G-F^ mice.

We next determined whether there was any selectivity for specific RGC subtypes in dendritic loss. RGCs can be categorised as ON-centre or OFF-centre, based on their response to illumination; these can be distinguished morphologically, ON-centre RGC dendrites projecting into sublamina b while OFF-centre RGC dendrites project into sublamina a [56]. In all three models, the loss of dendrites in ON-centred RGCs was more pronounced than in OFF-centre RGCs; ON-centre RGCs are depolarised by illumination. Their selective loss in the models may be a consequence of their higher energy demand and higher number of excitatory glutamatergic synapses [53].

Loss of hippocampal dendritic spines is a key hallmark of many AD models with spine loss particularly acute in the vicinity of amyloid plaques [46]. Dendritic spines can be subdivided into mushroom, stubby and thin categories based on morphology; [3] these morphological differences reflect functional diversity [39]. Thin spines are highly dynamic, capable of activity-dependent formation of new synapses during the process of learning. Loss of thin spines is associated with deficits in spatial learning and memory and has been reported in a range of CNS regions and contexts, such as the prefrontal cortex and hippocampus during normal ageing [57]. We used the SpineClassifier function in Imaris to label spine types on the basis of spine length and head size and re-analysed the hippocampal spine loss data. In each of the three AD models we observed a clear selective effect, a significant loss of thin spines with preservation of mushroom and stubby spines. Thin spine loss was particularly strongly correlated with the extent of RGC dendritic atrophy in the 3xTg-AD and APP^NL-G-F^ mice when compared with B6/J Controls. These findings reinforce the critical importance of dendritic spine loss as part of AD pathology; the observed thin spine selectivity fits with deficits in learning and memory in the models and in AD. Our results are supported by other studies that report dendritic spine loss, particularly in the hippocampus, of AD models [4, 35, 58]. The strong correlation with RGC degeneration indicates that retinal measures can provide an index of synaptic status in the hippocampus during AD pathology.

Gender variation in the 3xTg-AD model has been reported in the literature with more consistent pathology found in females [2]. We were able to separately measure RGC dendritic degeneration in male and female 3xTg-AD and APP^NL-G-F^ mice at 12 months; surprisingly, RGC dendritic loss was more pronounced in males in both models, more obviously in the 3xTg-AD model. Previous studies have reported other retinal pathologies in the 3xTg-AD model, including amyloid plaques, inflammation and microglial activation that could contribute to the observed RGC dendritic changes and gender differences in this model [13, 40]. The reason for these gender-dependent effects on RGC degeneration are unclear; however, given the known role of complement in synaptic loss in AD models [17, 43, 44, 55] and in synapse elimination in the CNS [48] we suggest that a possible driver of observed gender differences relate to differences in complement activity. Numerous studies have demonstrated gender differences in complement activity across mouse strains with male mice consistently showing more complement activity that females [24]; this would translate into more efficient complement activation at the synapse and greater synapse loss in male mice. Our (unpublished) analyses of the impact of complement gene deletion on synaptic loss in mouse AD models support this suggestion. Our study demonstrates the occurrence of RGC degeneration as an early and well-correlated marker of hippocampal neurodegeneration in both overexpressing and knock-in mouse models of AD. These findings provide vital evidence of significant retinal pathology in AD mouse models and reinforces the potential of monitoring disease by imaging the retina in human neurodegenerative diseases. There are limitations in extrapolating from models to human disease; hence it is possible that some of the reported retinal changes may not be reflected in the human disease [6]. Our demonstration that RGC dendritic degeneration occurs in the retina of knock-in APP^NL-G-F^ mice, the best available model for amyloidosis in the human disease because it does not suffer from overexpression artefacts, supports relevance. RGCs from APP^NL-G-F^ mice progressively degenerated with age and showed signs of dendritic changes early in the disease course that correlated with hippocampal changes and were in line with the known disease progression in the model.

Our data support the development of clinical imaging strategies targeting the inner plexiform layer (IPL), the region where RGC dendrites are located, to provide a non-invasive diagnostic tool and correlate of synaptic degeneration in the CNS in AD. RGC degeneration in the IPL can be imaged in vivo at high resolution using optical coherence tomography (OCT). With the development of broad-spectrum light sources, OCT can detect changes in the optical scattering characteristic of neurons undergoing degeneration and even changes in neuronal membrane refractive index that correlate with neuronal activity [21, 27, 34]. The demonstration that retinal changes occur in both the 3xTg-AD and APP^NL-G-F^ AD models early in the disease process and correlate with synaptic dysfunction, an early pathological event preceding neuronal cell loss and amyloid deposition in AD [9], indicates that retinal imaging has the potential to provide insights into the CNS health in mild cognitive impairment and the early stages of AD.

## Data Availability

The datasets used and/or analysed during the current study are available from the corresponding authors upon reasonable request.

## Abbreviations

AD: Alzheimer’s disease
CNS: Central nervous system
RGCs: Retinal ganglion cells
IPL: Inner plexiform layer
OCT: Optical coherence tomography
GCL: Ganglion cell layer
